# Genetic diversity and structure of the red squat lobster (*Grimothea monodon*) in the Humboldt Current Ecosystem using SNP markers

**DOI:** 10.7717/peerj.20580

**Published:** 2026-01-13

**Authors:** Marco Quispe-Machaca, Carlos Muñoz-Ramírez, Antonio Brante, Pepe Espinoza, Maximiliano Zilleruelo, Gabriela Torres, Ángel Urzúa

**Affiliations:** 1 Programa de Doctorado en Ciencias mención Biodiversidad y Biorecursos, Facultad de Ciencias, Universidad Católica de la Santísima Concepción, Concepción, Biobío, Chile; 2 Instituto de Entomología, Universidad Metropolitana de Ciencias de la Educación, Santiago, Santiago, Chile; 3 Departamento de Ecologia, Facultad de Ciencias, Universidad Católica de la Santísima Concepción, Concepción, Biobío, Chile; 4 Centro de Investigación en Biodiversidad y Ambientes Sustentables (CIBAS), Universidad Católica de la Santísima Concepción, Concepión, Biobío, Chile; 5 Instituto del Mar del Perú (IMARPE), Esquina Gamarra y Gral, Lima, Peru; 6 Facultad de Ciencias Biológicas y Veterinarias, Carrera de Biología Marina, Universidad Cientifica del Sur, Lima, Peru; 7 Instituto de Fomento Pesquero (IFOP), Valparaiso, Valparaiso, Chile; 8 Alfred-Wegener-Institut für Polar und Meeresforschung, Helgoland, Schleswig-Holstein, Germany

**Keywords:** Marine invertebrates, Morphotypes, Humboldt Current Ecosystem, Genetic variability, Molecular markers, Single nucleotide polymorphism, Crustaceans

## Abstract

The Humboldt Current Ecosystem (HCE) presents a wide variability of environmental and geographic conditions that play an important role in marine invertebrates, modulating variations not only in their behavior, physiology and morphology; but also changes in their patterns of genetic differentiation. Therefore, it is necessary to characterize genetic diversity and structure to develop management and conservation strategies in commercially important invertebrates such as the red squat lobster *Grimothea monodon*, which in the HCE presents two highly contrasting morphotypes and/or lifestyles (pelagic *vs.* benthic). The objective of the present study was to evaluate the diversity, structure, and genetic connectivity of the *G. monodon* population along the latitudinal gradient in the HCE using single nucleotide polymorphism (SNP) markers. Low heterozygosity (0.05 ± 0.1) and similar allelic richness among all studied populations (∼1.09) were observed. However, a slightly higher inbreeding was recorded in individuals from the Concepción population. At the level of genetic structure, using LEA and STRUCTURE, it is confirmed that *G. monodon* is a single population unit between the pelagic and benthic morphotypes, and that the difference observed in the discriminant analysis of principal components (DPCA) is due to the geographic distance (isolation by distance) between the extreme southern locations of the HCE (Constitución-Concepción: verified by Analysis of Molecular Variance (AMOVA) and Mantel test). The slight difference observed mainly in Concepción is due to inbreeding, however, this tends to be very low due to the high genetic flow explained by the prolonged development time of their planktonic larvae, which can positively influence an optimal recovery of their natural populations along the latitudinal gradient. This study emphasizes that the slight genetic differentiation of *G. monodon* could be due only to its wide geographic distribution range, generating only intra-population variability and not inter-population variability. This is due to the high dispersal potential of their planktonic larvae, which converges as a development trait of early ontogeny for both morphotypes and/or lifestyles of this squat lobster.

## Introduction

Understanding the patterns of population structure and differentiation in marine invertebrates of fishing importance with a wide distribution range is important for the conservation, management and sustainable exploitation of these marine bioresources (Lowell et al., 2023). In this context, at the genetic level, the diversity and structure of populations can reflect both the influence of environmental factors and the biology of species (Bert et al., 2014; Silva & Gardner, 2016; Boulanger et al., 2022). In the case of marine invertebrates that have a wide ecological niche (*i.e.,* realized niche) in habitats with variable environmental conditions, their dispersal potential and/or connectivity can vary along a latitudinal gradient (Einfeldt, Zhou & Addison, 2017). Habitat geography also plays an important role in generating patterns of genetic differentiation (Kimura & Weiss, 1964; Duforet-Frebourg & Slatkin, 2015), where biogeographic barriers that promote local environmental conditions can influence genetic divergence between and within populations (Tovar Verba et al., 2022). In addition, it has recently been described that the influence of anthropogenic disturbances can also alter the genetic structure and spatial distribution of species in their natural environment (Chiu et al., 2023).

Within a marine ecosystem, there are intrinsic and extrinsic factors that can influence the dispersal capacity of species, such as the larval development time and the presence of biogeographic barriers, which consequently affect the genetic connectivity of populations (Kelly & Palumbi, 2010; Haye et al., 2014). In turn, these factors can be attenuated or pronounced by environmental conditions. For example, sea temperature can influence the metabolism, growth and survival of larvae, while ocean currents can affect their movement through plankton and their ability to colonize new habitats (Wares, Gaines Steven & Cunningham, 2001). In marine invertebrates with a complex life cycle (for a concept see: Wilbur (1980), Moran (1994) and Formery & Lowe (2023)), the dispersal ability of larvae is highly relevant because it drives important demographic and evolutionary processes by promoting population connectivity (Chiu et al., 2023). In this way, competition and inbreeding are minimized, thus allowing larvae to colonize new habitat patches and potentially generate new populations (Suárez et al., 2022). In addition, high gene flow allows populations to maintain more stable population sizes, reducing the rate of extinction and speciation (Haye et al., 2014).

There are a variety of molecular markers that allow us to understand the diversity and genetic structure of marine populations (*e.g.*, Cytochrome c Oxidase subunit I (COI), Amplified Fragment Length Polymorphism (AFLP)). These markers have been used to study the genetic structure and connectivity of populations of marine organisms with complex life cycles, such as macroalgae (Tellier et al., 2009; Fraser et al., 2010), invertebrates (Haye et al., 2014; Haye et al., 2019; Ewers-Saucedo et al., 2016; Quesada-Calderón et al., 2021) and fish (González-Wevar et al., 2015). Currently, population genetic studies are gaining more relevance through molecular markers such as single nucleotide polymorphism (SNP), which has obtained denser genotypes with greater representation of the species genome (Uptmoor et al., 2003; de Villemereuil et al., 2016). Unlike mitochondrial markers (*e.g.*, mtDNA COI, Cyt b), SNP markers utilize adaptive loci (outlier loci) to aid in confirming whether there is a weak or null genetic structure within a certain population at a variety of spatial and environmental scales (Jenkins et al., 2019), as has been reported in species with low dispersal potentials, such as *Pyure chilensis* (Segovia et al., 2017) or high dispersal potentials, such as *Metacarcinus edwardsii* (Veliz et al., 2022).

The Humboldt Current Ecosystem (HCE) is one of the most important systems worldwide due to its wide variety of environmental and oceanographic conditions (Carrasco et al., 2021) and the presence of topographic discontinuities (Rodríguez-Romero et al., 2022), which generate “barriers” or “biogeographic breaks” that establish complex environments and a mosaic of habitats (Berend, Haynes & MacKenzie, 2019). These barriers have an impact on the connectivity of natural populations. Within the HCE, known biogeographic breaks are located between 5°S–6°S and 12°S (Ibanez-Erquiaga et al., 2018), 30°S (Brante, Fernández & Viard, 2012; Segovia et al., 2017; Weidberg et al., 2020), and 42°S (Fernandez et al., 2000; Leclerc et al., 2018). A transition break between 24°S–25°S has also been described along the coasts of Chile, although some authors do not consider this of relevance (Ojeda, Labra & Muñoz, 2000; Haye et al., 2010; Sielfeld et al., 2010; Navarrete, Lagos & Ojeda, 2014). Nonetheless, the changes observed in richness and abundance of marine species and assemblages coincide with this biogeographic break (Navarrete, Lagos & Ojeda, 2014). For example, changes in the morphological traits of some marine species, such as the red squat lobster *Grimothea monodon* (previously known as *Pleuroncodes monodon* (Baba et al., 2008), have been recorded (Machordom et al., 2022).

*Grimothea monodon* (Decapoda: Munididae) supports the main industrial fishery of demersal crustaceans in the Southeastern Pacific Ocean. Its geographic distribution covers a large part of the Humboldt Current Marine Ecosystem (Perú-Chile) from Isla Lobos de Afuera in Perú to Chiloé in Chile (Hendrickx & Harvey, 1999; Hernáez & Wehrtmann, 2011). *G. monodon* presents two morphotypes: (i) pelagic (5°S–24°S) and (ii) benthic (∼24°S to 48°S) (Haye et al., 2010). Currently, its distribution range has been extended to North America (DecaNet, 2024). In this context, its wide distribution could be explained by its high fecundity and dispersal potential, characterized by a large egg production (approx. 1,808–33,966 embryos), from which numerous planktonic larvae hatch and live in the water column for ca. 90 days (Palma & Arana, 1997; Hernáez & Wehrtmann, 2011). Through a single molecular analysis with the mitochondrial marker COI, it was determined that the pelagic and benthic morphotypes of *G. monodon* correspond to a single genetic population, suggesting a heterochronic phenotypic plasticity that is expressed during its early ontogeny (Haye et al., 2010).

Environmental factors, such as temperature, influence generation time, mutation rates and physiological processes (growth and reproduction) (Willig & Presley, 2018) of ectothermic marine animals (Sunday, Bates & Dulvy, 2012; Weber et al., 2015) with high thermal (Bennett et al., 2019) and physiological sensitivity (Sunday, Bates & Dulvy, 2011; Sunday, Bates & Dulvy, 2012). In marine environments, variations in temperature along a latitudinal gradient can exert a potential selective pressure that modifies the phenotypic responses of a population with a wide distribution range (Lardies, Arias & Bacigalupe, 2010; Barria et al., 2018), resulting in a phenotypic plasticity response (Acasuso-Rivero et al., 2019; Burton, Ratikainen & Einum, 2022), which can be reversible or irreversible (Forsman, 2015; Futuyma & Kirkpatrick, 2017; Hoffmann & Bridle, 2022). Consequently, the ability of a population to adapt to a heterogeneous environment depends on the changes that occur in its physiological traits and its life history (thermal sensitivity, metabolic rate, fecundity) (Nilsson, 2012; Calosi et al., 2013).

In turn, other evolutionary scenarios could also explain the presence of distinct phenotypes in different areas (Rutschmann et al., 2016; Vinton et al., 2022). For example, the presence of barriers to gene flow (*i.e.,* biogeographic barriers) could generate a cessation of gene flow between populations, triggering the morphological differentiation of adult individuals by both neutral (genetic drift) and selective mechanisms acting independently in isolated populations (Malvé, Rivadeneira & Gordillo, 2018). Although a high gene flow in *G. monodon* has been suggested throughout its distribution based on mitochondrial sequences (Haye et al., 2010), it remains to be evaluated whether this apparent pattern is supported by analyzing the genetics of the populations in more recent conditions with a more informative marker (*e.g.*, SNPs). This can be explained by the fact that mitochondrial markers have a single locus due to the lack of recombination and can thus provide a biased view of the level of differentiation or be exposed to incomplete lineage sorting processes (Hurst & Jiggins, 2005; Bradman, Grewe & Appleton, 2011).

To better understand the evolutionary ecology of the red squat lobster, nuclear markers with higher resolution power must be utilized (Morin et al., 2004; Wenne, 2023). Therefore, the objective of the present study was to evaluate the diversity, connectivity and genetic structure of the pelagic and benthic populations of *G. monodon* throughout its wide latitudinal distribution range, and to test its correlation with biogeographic barriers using SNP markers. We hypothesized that the pelagic and benthic morphotypes of *G. monodon* distributed along the latitudinal gradient of the HCE present a genetic structure concordant with the distribution of its morphotypes.

## Materials & Methods

### Collection of samples

**Figure 1:**
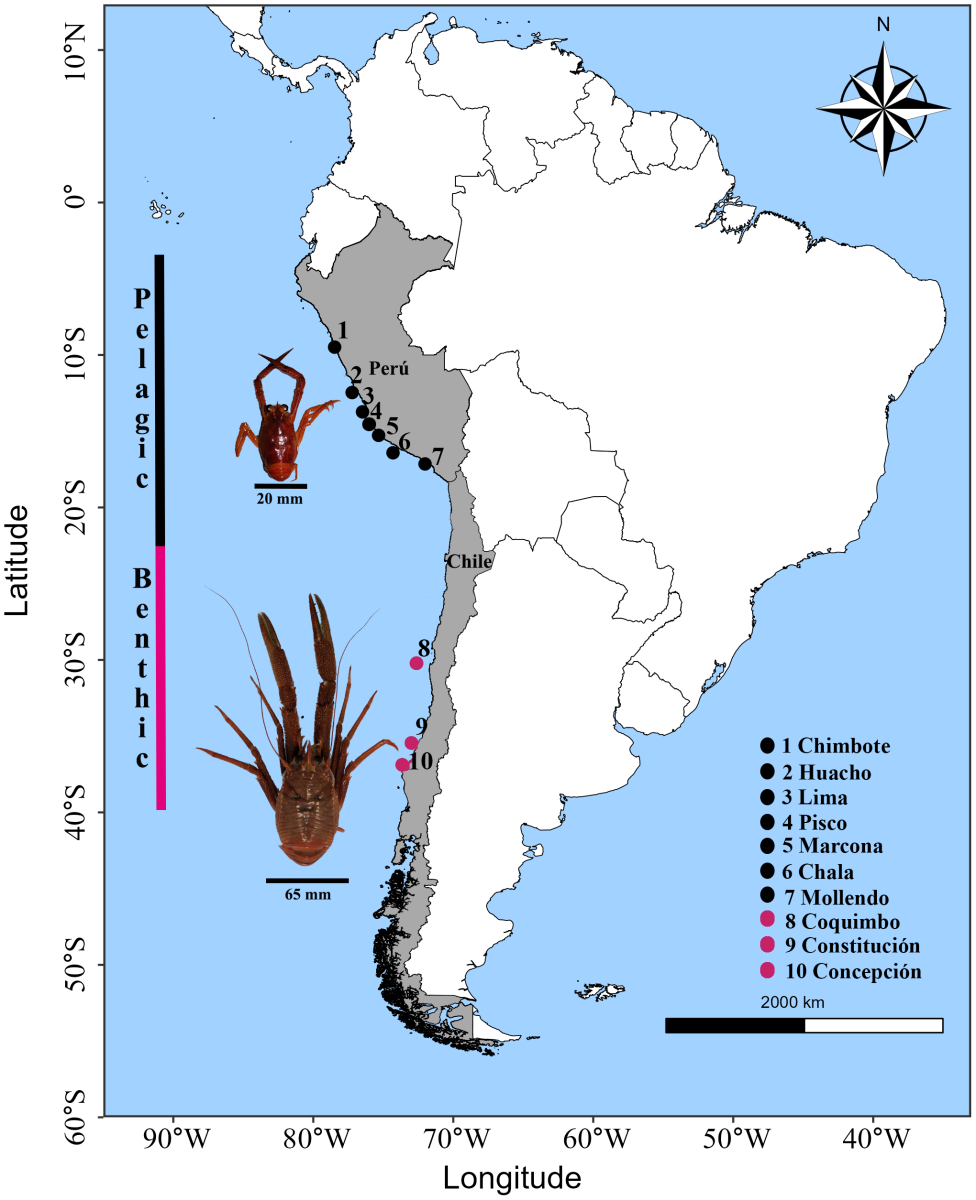
Sampling locations of the red squat lobster *Grimothea monodon* off the coast of Perú and Chile in the Humboldt Current Ecosystem. Horizontal lines in black represent the size (cephalothorax length) in each morphotype. Vertical lines represent the distribution range in each morphotype.

Adult individuals of *G. monodon* were collected in the southeastern Pacific Ocean within the Humboldt Current Ecosystem. The pelagic morphotype was collected by the Peruvian Sea Institute (IMARPE) in Peru through the monitoring of anchovy fisheries as by-catch, and the benthic morphotype was obtained through the monitoring of the demersal crustacean fishing fleet by the Fisheries Development Institute (IFOP) in Chile. The sampling range in the HCE covered an extension of ca. 6,000 km from 9°S latitude to 36°S latitude, corresponding to a total of 10 locations where the pelagic (09°S–17°S; Peru) and benthic (30°S–36°S; Chile) morphotypes were obtained (Fig. 1; Table 1). For each sampling location, *n* = 10 individuals were processed, except for Pisco (*n* = 4 individuals), with a total of *n* = 94 samples. Only muscle tissue samples from the chelae were extracted from each individual; these were placed in a PCR tube with 99% pure alcohol. The prepared samples were sent to the Diversity Array Technology (DArT) laboratory in Australia. The DNA was extracted, sequenced, and the informative SNP markers were processed (https://www.diversityarrays.com, Kilian et al., 2012) for the elaboration of the DarTSeq libraries (Wenzl et al., 2004). The DarTSeq method obtains high-performance markers of the entire genome by reducing the complexity of the genome mediated by restriction enzymes (Edet et al., 2018). This tool is ideal for characterizing the genome of “species without genomic reference” or other previous information (Ayllón, 2017).

### Genetic data processing

The raw sequence data (accession number PRJNA1242819) is available at https://www.ncbi.nlm.nih.gov/bioproject/PRJNA1242819/. To construct the de novo genetic map of the samples, the DartR program (Kilian et al., 2012, http://georges.biomatix.org/dartR), a software package that operates in R (R Core Team, 2024), was used. The data obtained by DArTSeq contained associated metadata (CloneID, Callrate, SnpPosition, AvgPIC), which were used for further filtering. The SNP data were loaded as a genlight object in R, and filtering was applied to remove: (i) unreliable loci (*i.e.,* those with exceptionally low or high read depths), (ii) loci with low allele frequencies (>5%), and (iii) loci with high levels of missing data (Gruber et al., 2018). Specifically, loci with more than 88% missing data were excluded, and the 20% of individuals (*N* = 12) with the highest proportion of missing data were also removed. Finally, any remaining monomorphic loci were discarded.

### Genetic diversity, structure and connectivity

To study genetic diversity, genetic parameters such as observed heterozygosity (Ho), expected heterozygosity (He), inbreeding (FIS) and allelic richness (AR) were evaluated in each biogeographic area. As a first step, and in an exploratory manner, a principal component analysis (PCoA) and a discriminant analysis of principal components (DAPC) were performed, which identified and described genetically related groups (Jombart, Devillard & Balloux, 2010). Then, an admixture analysis using sparse nonnegative matrix factorization was conducted in Landscape and Ecological Association (LEA package) (Frichot & François, 2015) to evaluate the population genetic structure. In turn, to validate the biostatistical method, a Bayesian analysis of genetic structure implemented in the STRUCTURE v2.3.4 program was performed (Pritchard, Stephens & Donnelly, 2000). These analyses evaluate cluster-based genetic differentiation under the assumption of Hardy-Weinberg transient disequilibrium (HWD) and linkage disequilibrium (Kaeuffer et al., 2007). Subsequently, a molecular analysis of variance (AMOVA) was performed to assess the genetic variability between the morphotypes in the different localities (Excoffier, Smouse & Quattro, 1992). The differentiation between populations was assessed using the *F*_st_ test (Weir & Cockerham, 1984). The transfer of genetic material (gene flow) between localities was indirectly calculated from the *F*_st_ (Wright, 1969; Slatkin, 1987), and the Mantel correlation test was performed through the isolation by distance (IBD) test to determine the influence of geographic distance on gene flow (Diniz-Filho et al., 2013). Finally, to evaluate the possibility of unidirectional dispersal—given that the species has a pelagic stage and its dispersal may be influenced by ocean currents (Cowen & Sponaugle, 2009)—asymmetrical migration between sites was estimated using divMigrate-online (Sundqvist et al., 2016). This program statistically infers differences in the magnitude of gene flow between sites using distance metrics that are not biased by genetic diversity. For this analysis, the genlight object was converted into a genepop file using the function gl2genepop available in the R package adegenet (Jombart, 2008; Jombart & Ahmed, 2011). Gene flow was estimated using the D statistic (Jost, 2008; Zheng & Janke, 2018), and statistical support was assessed with 200 bootstrap replicates.

**Table 1:** Genetic diversity of the red squat lobster (*G. monodon*) across its two morphotypes along the latitudinal gradient of the Humboldt Current Ecosystem.

**Morphotype**	**Locality**	**N**	**Latitude**	**Longitude**	**Ho**	**HoSD**	**He**	**HeSD**	**FIS**	**Ar**	**Priv**	**Fix**
**Pelagic**	**Chimbote**	8	09°29.1′0.00″S	78°28.6′0.00″W	0.03	0.09	0.05	0.10	0.29	1.09	1649.11	0
	**Huacho**	6	11°19.4′0.00″S	78°17.6′0.00″W	0.04	0.10	0.05	0.10	0.20	1.09	1324.9	0
	**Lima**	10	12°27.8′0.00″S	77°13.5′0.00″W	0.04	0.09	0.05	0.10	0.24	1.09	2173.6	0
	**Pisco**	4	13°43.0′52.0″S	76°28.0′59.0″W	0.04	0.11	0.04	0.11	0.25	1.08	880.7	0
	**Marcona**	10	15°16.3′0.00″S	75°20.2′0.00″W	0.04	0.09	0.04	0.09	0.15	1.09	2097	0
	**Chala**	9	16°24.9′0.00″S	74°18.4′0.00″W	0.04	0.09	0.05	0.10	0.30	1.10	1992.11	0
	**Mollendo**	10	17°8.80′0.00″S	72°1.10′0.00″W	0.03	0.08	0.04	0.09	0.28	1.08	1912.9	0
**Benthic**	**Coquimbo**	9	30°12′52.16″S	72°37′33.20″W	0.04	0.08	0.05	0.10	0.30	1.10	2010.4	0
	**Constitución**	9	35°26.0′53.0″S	72°59.0′13.0″W	0.04	0.09	0.05	0.10	0.30	1.10	1952.9	0
	**Concepción**	6	36°52.0′50.0″S	73°38.0′55.0″W	0.03	0.09	0.05	0.11	0.32	1.09	1231.4	0

**Notes.**

Ho Observed heterozygosity HoSD Standard deviation of observed heterozygosity He Expected heterozygosity HeSD Standard deviation of expected heterozygosity FIS Inbreeding coefficient Ar Allelic richness Priv Mean number of private alleles per population pair Fix Mean number of fixed alleles per population pair

## Results

From a total of *n* = 94 individuals collected in 10 localities in Peru and Chile, a total of *n* = 90,539 SNPs were obtained. Subsequently, after filtering, a total of 12,616 SNPs for 81 individuals were available for downstream analyses. For details regarding the individuals and localities used in analyses, see Table 1.

### Genetic diversity

The genetic diversity (observed and expected) for the 10 studied locations was low. The heterozygosity index (He) among populations varied between 0.04 in Pisco (*n* = 4) and 0.05 in Coquimbo (*n* = 9), with a total average of 0.037. The Ho varied between 0.033 in Mollendo (*n* = 10) and 0.04 in Marcona (*n* = 10), with a total average of 0.037. Allelic richness (AR) was similar among all populations evaluated along the latitudinal gradient, with observed values between 1.08−1.10 (Table 1). Regarding the inbreeding index (FIS), this was very low, with a total recorded value of 0.28 along the latitudinal gradient. When evaluating inbreeding in each locality, the locality of Marcona showed the lowest inbreeding index (FIS= 0.1485), while the highest inbreeding value recorded was 0.32 in the locality of Concepción (Table 1).

### Genetic structure of evaluated populations

**Figure 2:**
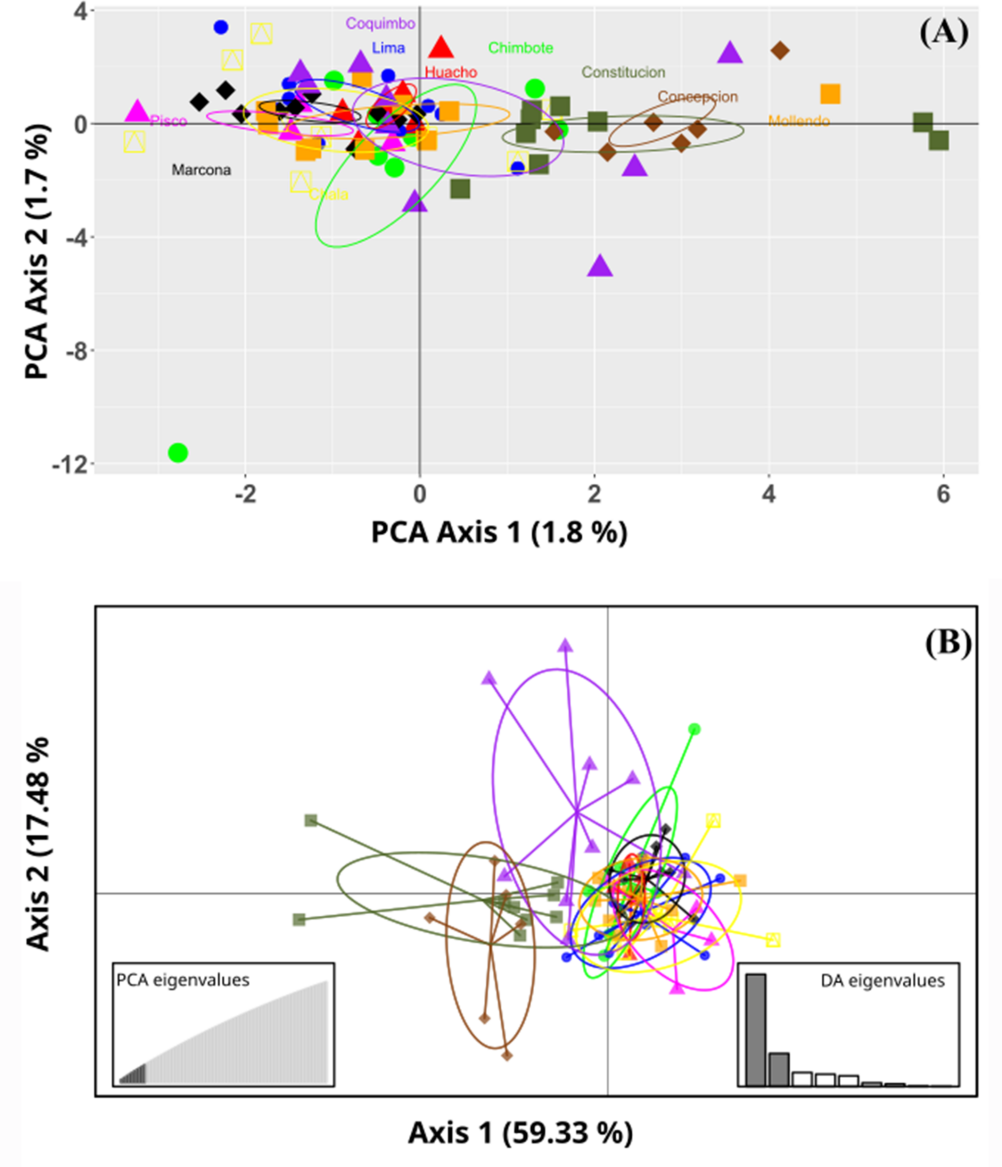
Ordination plots of the *Grimothea monodon* individuals. (A) Principal component analysis (PCA) and (B) Discriminant analysis of principal components (DAPC). (A) PCA, the axes represent the percentage of variability explained by each principal component, each of the symbols represents the individuals in each population. (B) DAPC, the axes represent the percentage of variability explained by each coordinate. The circles represent the 95% confidence limit, and the points represent the individuals in each population.

No marked genetic structures were found in the analysis of the evaluated populations. The principal component analysis (PCoA) revealed a slight separation between the benthic morphotypes from the Constitución (35°S) and Concepción (36°S) localities. In turn, all the sampled populations of the pelagic morphotype were grouped together with the benthic population from the Coquimbo locality (30°S). The total explained variability was 3.5% in the PC1 and PC2 components. This result was similar to that of the Discriminant Analysis of Principal Components (DAPC); here, the optimal cluster value “k” obtained by the silhouette method was *K* = 2. In contrast, the Gap statistical analysis for the real optimal cluster value was *K* = 1. The DAPC presented a similar result to the PCoA; here, the explained variability represented 76.81%, where the benthic morphotypes from the locations of Constitución and Concepción were classified as a single group and were clearly separated from those from the Coquimbo population. Consequently, these were noticeably different from the pelagic morphotype group (Fig. 2).

**Figure 3:**
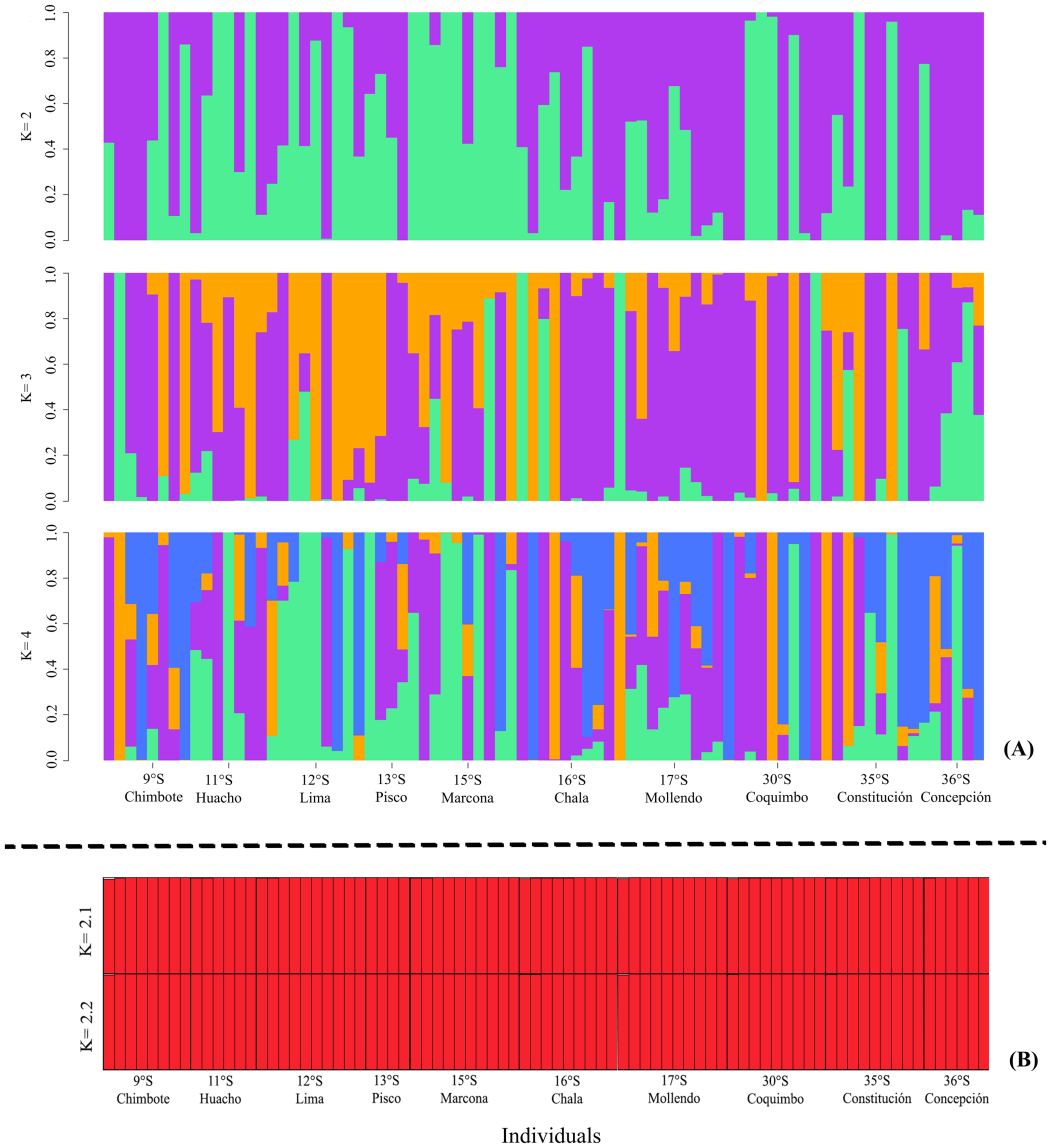
Diagrams of the genetic structure of the red squat lobster *Grimothea monodon* using neutral loci. (A) Structure analysis performed with ADMIXTURE in LEA. (B) Bayesian analysis of genetic structure.

The ADMIXTURE in the LEA analysis showed no genetic structure in the *K* = 2, *K* = 3, *K* = 4 bar plots. This result was similar to that observed in STRUCTURE, where *K* = 2 best represented the biological analysis (Fig. S1) of the genetic structure of the evaluated populations (Fig. 3). Through the fixation index (*F*_st_), it was observed that *G. monodon* presented a low genetic differentiation between the different populations and localities (*F*_st_ = 0.0026). This result was verified through the pair test between the different populations (Table 2), where the greatest genetic divergence was observed between the localities of Constitución-Concepción and Marcona, with a *F*_st_ of 0.0109 and 0.0179, respectively. Consistently, a low dispersion potential was recorded in the comparisons Marcona ^x^ Constitución (22.64) and Marcona ^x^ Concepción (13.74) (Table 2).

### Genetic connectivity

**Figure 4:**
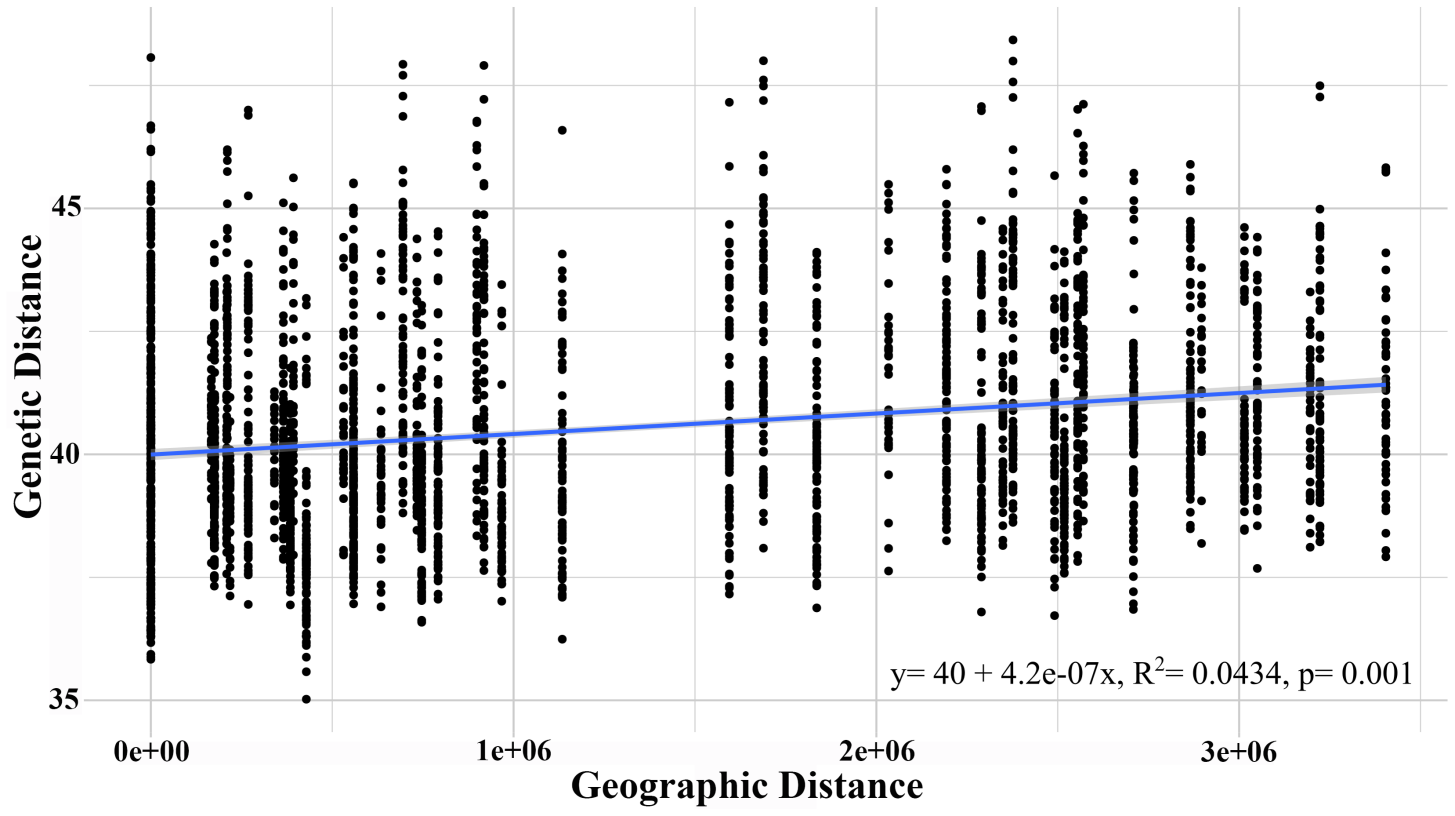
Isolation by distance analysis (IBD), describing the correlation between genetic distance and geographic distance.

The AMOVA test with a significance level of 95% revealed that the highest genetic variation was recorded within the sampled individuals (71.73%) and between individuals within each population (27.71%), though both presented a low fixation index. Although the *G. monodon* morphotypes presented a low percentage of genetic variation, significant statistical differences were found (*p* = 0.01) (Table 3). This finding was similar to that of the isolation by distance analysis, which showed significant differences through the Mantel test (*R*^2^ = 0.198, *p* = 0.016) (Fig. 4). Regarding asymmetrical migration, the divMigrate-online analysis provided no evidence of unidirectional gene flow between any pair of populations (see Table S2).

## Discussion

The HCE is characterized by its environmental mosaics of contrasting physical-chemical factors (*e.g.*, temperature, nutrients, upwelling) and biogeographic breaks or environmental barriers along a latitudinal gradient; all of these features consequently influence the structure, composition, and genetic diversity of marine organisms (McClain & Hardy, 2010; Sánchez et al., 2011; Heads, 2015; Berend, Haynes & MacKenzie, 2019). The great variability in habitat conditions modulates the gene flow in species with wide biogeographic distributions, as reported in *G. monodon* (Yapur-Pancorvo et al., 2023) and also in the marine invertebrates: *Liocarcinus depurator* (Ojeda et al., 2022), *Tegula atra*, *Scurria scurra*, *Emerita analoga*, and *Heliaster helianthus* (Haye et al., 2014), fish: *Xiphias gladius* (Lu et al., 2016; Grewe et al., 2020), and algae: *Macrocystis pyrifera* (Iha et al., 2023). All of the aforementioned marine organisms have a complex life cycle characterized by a pelagic larval phase with the dispersal potential to colonize new habitats and/or allow flow between populations. In this context of connectivity and structure, some studies in the HCE indicate that a strong signal of genetic structure in many marine species may be influenced by the presence of biogeographic breaks (*e.g.*, off the coast of Chile at 30°S) where important oceanographic and physical changes have been reported as upwelling areas, thermal discontinuities, scarce to narrow continental shelf (Deville et al., 2021). The presence of a notable genetic structure occurs mainly in species that present low dispersal potential, as reported by Haye et al. (2014), who indicated that low genetic diversity is linked to the effects of genetic drift of species (Kelly & Palumbi, 2010).

**Table 2:** Pairwise estimates of F_ST_ (lower diagonal) among populations of the red squat lobster (*G. monodon*) from the coasts of Peru and Chile in the Humboldt Current Ecosystem.

** **	**Chimbote**	**Huacho**	**Lima**	**Pisco**	**Marcona**	**Chala**	**Mollendo**	**Coquimbo**	**Constitución**
**Chimbote**									
**Huacho**	0.0024								
**Lima**	0.0030	0							
**Pisco**	0	0.0002	0						
**Marcona**	**0.0071**	**0.0058**	**0.0038**	**0.0085**					
**Chala**	0	0	0	0	**0.0062**				
**Mollendo**	0.0006	0.0014	0.0011	0.0013	**0.0037**	0			
**Coquimbo**	0.0034	0	**0.0035**	0	**0.0075**	0.0009	**0.0028**		
**Constitución**	**0.0053**	0.0039	**0.0058**	0.0010	**0.0109**	**0.0045**	**0.0072**	**0.0038**	
**Concepción**	**0.0062**	**0.0051**	**0.0072**	0.0018	**0.0179**	**0.0043**	**0.0105**	**0.0047**	0

**Notes.**

Significant values at the 95% confidence level are shown in bold.

**Table 3:** Hierarchical molecular analysis of variance (AMOVA), showing the degree of differentiation between and within individuals captured along the latitudinal gradient in the Humboldt Current Ecosystem.

** **	**Df**	**Sum Sq**	**Mean Sq**	**Sigma**	**%**	**Phi**	***P* value**
**Variation between morphotype**	1	1,022.21	1,022.21	2.77	0.43	0.0033	0.01
**Variation between populations within morphotype**	8	6,673.48	834.19	0.85	0.13	0.0013	1.00
**Variation between individuals within population**	71	58,262.97	820.61	178.82	27.71	0.2786	0.00
**Variation within individuals**	81	37,499.77	462.96	462.96	71.73	0.2810	
**Total**	161	103,458.43	642.60	645.40	100.00		

**Notes.**

Df Degree of freedom

The study of genetic diversity is important for the development of strategic plans to manage and conserve important marine species (Oñate, 2012). For example, identifying the heterozygosity index is important to assess population variation at the genetic level, and the observed heterozygosity is often influenced by sample size (Qin, Shi & Sun, 2013; Duan et al., 2022). In populations of *G. monodon*, no variation in genetic diversity was detected across the study range based on any of the diversity indices. Allelic richness is considered a good indicator of genetic diversity compared to heterozygosity, as well as being considered an important indicator of selection potential (Greenbaum et al., 2014). The generally high allelic richness observed in most populations provides no clear evidence of inbreeding in southern populations, contrary to previous assumptions based on the strong exploitation of other marine resources (Pinsky & Palumbi, 2014; Sadler, Watts & Uusi-Heikkilä, 2023).

Despite evidence of overexploitation of *G. monodon*, which led to the collapse of the benthic population in the Southern Fishing Unit (SFU) (Yapur-Pancorvo et al., 2023; Barros et al., 2024), our data do not provide conclusive evidence for a reduction in genetic diversity or an increase in inbreeding in southern populations. This may be explained by the implementation of fishing bans along the Chilean coast (Queirolo et al., 2015) and/or by extensive population mixing driven by high levels of gene flow. Moreover, based on multiple analyses of genetic structure (*e.g.*, STRUCTURE, DAPC, IBD), we detected low levels of differentiation among populations. This finding indicates the existence of one single genetic population, which is in accordance with the results of a mitochondrial analysis reported by Haye et al. (2010). Although the Principal Coordinate Analysis (PCoA) and the Discriminant Analysis of Principal Components (DAPC) revealed slight structuring—where the clustering of samples or individuals corresponded to their geographic distribution in the HCE (Barros, Alarcón & Arancibia, 2023)—this weak geographic signal may reflect isolation by distance, as confirmed by the Mantel test. The overall lack of strong genetic structure, indicative of high gene flow, could explain the similar levels of genetic diversity observed in *G. monodon* along the Peruvian and Chilean coasts.

Given the lack of population genetic structure found for the species, which has been also reported by Haye et al. (2010), it remains unclear how the marked morphological differences between morphotypes are maintained despite high levels of gene flow. Haye et al. (2010) proposed that the variability in the morphology of *G. monodon* could be explained by phenotypic plasticity as a response to the environmental conditions that predominate during its early ontogeny. In this context, adult *G. monodon* have different lifestyles and/or morphotypes (pelagic *vs.* benthic) throughout their latitudinal distribution range in the HCE, probably due to the variability of the environmental conditions and geomorphological characteristics of the habitat, such as the presence of the continental shelf (Deville et al., 2021). Therefore, the absence of a distinct genetic structure for the two morphotypes is due to the high dispersal potential of their pelagic larvae, which converge as a development trait of early ontogeny for both morphotypes and/or lifestyles of this species. This pelagic larval phase of *G. monodon* lasts ca. 90 days in the water column (Fagetti & Campodonico, 1971; Yannicelli et al., 2013; Á Urzúa, pers. obs., 2015), a period which most likely allows connectivity to be maintained between the two populations along the latitudinal gradient. In turn, the high larval dispersal potential presented by *G. monodon* is not limited by the large biogeographic break that occurs at 30°S or by important environmental barriers, such as upwellings that vary in intensity off the coasts of Peru, northern and south-central Chile (Montecino & Lange, 2009; Deville et al., 2021), as has been observed in other crustaceans such as *Metacarcinus edwarsii* (Veliz et al., 2022).

On the other hand, the clustering of the *G. monodon* populations in our results reflects their geographic distribution, suggesting some role of geographic distance as was observed in genus *Munida* (Wang & Bradburd, 2014; Yan et al., 2020). This result was similar to the one observed for the species *Stichaster striatrus*, which has also shown a high potential for larval dispersal, a degree of isolation by distance, and a weak genetic structuring (Haye et al., 2014). The genetic differentiation presented by the red squat lobster, mainly due to the geographic distance between the most extreme study locations (Chimbote (9°S) *vs.* Concepción (36°S), with an approximate distance of 3,500 km between them), could indicate the presence of a slight genetic heterogeneity in this species in relation to its geographic distribution, similar to that reported by Ellis et al. (2017) in the lobster *Homarus gammarus*. This species of lobster, like the red squat lobster *G. monodon*, shares attributes of commercial importance, overexploitation by fisheries and a wide distribution (Hinchcliffe et al., 2022; Jurrius & Rozemeijer, 2022). In agreement with the above, and particularly with the high genetic flow that *G. monodon* has presented, our findings suggest that the pattern observed for the red squat lobster fits the stepping-stone model (Kimura & Weiss, 1964) that is influenced by the Humboldt Current from 42°S northwards.

The importance of exploring genetic diversity and structuring lies in determining whether any adaptive process is being generated in response to important environmental changes (Harvey et al., 2014; Chung et al., 2023). Genetic diversity increases chances of survival and of obtaining new phenotypic traits to face changes in environmental or anthropic factors (Alcala et al., 2013), such as the two morphotypes or phenotypes that *G. monodon* presents in the HCE. During closed season this resource may be able to migrate from north to south thanks to its high genetic variability, which is consistent with the high gene flow between Huacho and Pisco (Nm = 1,627 ind/gen). In this sense, a high gene flow between individuals of the pelagic *vs.* benthic morphotypes was also revealed between the locality of Pisco, Constitución (Nm = 241 ind/gen) and Concepción (Nm =1 39 ind/gen); In addition, the gene flow between benthic individuals from the locations of Constitución and Concepción was high, with an infinite value of genetic flow. A similar response has been described for other species of decapods with a wide distribution range in temperate and cold coastal regions (Hudson, Slatkin & Maddison, 1992; Yuan et al., 2010).

In turn, the migratory behavior of *G. monodon* can help to recover its population stock and maintain ecosystem balance, particularly for the stability of marine food webs where it plays an important role in the transfer of energy in biogeochemical processes of benthic-pelagic coupling (Guzmán-Rivas et al., 2021). As revealed in this study, *G. monodon* presents a high resilience to negative impacts (natural or anthropogenic), a high genetic flow and low genetic structuring. All these characteristics could allow both pelagic and benthic morphotype individuals to be maintained and bred under captive conditions. Thus, larval culture technologies could help repopulate this species by releasing captive-bred juveniles into geographic areas where a significant population decline has been reported due to overfishing.

## Conclusions

Finally, according to the analyses carried out in this study using SNP markers, we can conclude that biogeographic barriers are not a limiting factor in the structuring and genetic diversity of the red squat lobster *G. monodon*. The life-history characteristics of this species (morphotypes: pelagic *vs.* benthic) in its adult stages did not represent genetic breaks, it could indicate that these morphotypes have not originated from neutral evolution and a lack of genetic flow. The most important trait that influences the low structuring and genetic variability could be the pelagic larval phase, which is present and converges in both morphotypes. These larvae remain in the plankton for a long time before their metamorphosis to the megalopa stage and subsequent first juvenile, which depending on their ability to settle on the bottom or swim in the open ocean could determine the type of lifestyle of the adults (benthic or pelagic, respectively).

##  Supplemental Information

10.7717/peerj.20580/supp-1 Supplemental Information 1 Genetic Sequences of *Grimothea monodon*

10.7717/peerj.20580/supp-2 Supplemental Information 2 Fixed and private Alleles

10.7717/peerj.20580/supp-3 Supplemental Information 3 Relative directional Migration matrix of gene flow between *G. monodon* populations

10.7717/peerj.20580/supp-4 Supplemental Information 4 Evanno plot of the STRUCTURE analysis indicating the number of optimal number of cluster (K)

## Additional Information and Declarations

### DNA Deposition

The following information was supplied regarding the deposition of DNA sequences: The genetic sequences of Grimothea monodon is available at NCIB: PRJNA1242819.

### Data Availability

The following information was supplied regarding data availability: The raw data is available in the Supplemental Files and Zenodo: Quispe-Machaca, M., Muñoz-Ramírez, C., Brante, A., Espinoza, P., Zilleruelo, M., Torres, G., & Urzúa, Á. (2024). Diversity and genetic structure of the red squat lobster (*Grimothea monodon*) in the Humboldt Current Ecosystem using SNP markers [Data set]. Zenodo. https://doi.org/10.5281/zenodo.15191197.
