# Supplementary figures and images for: Genetic diversity and structure of the red squat lobster (Grimothea monodon) in the Humboldt Current Ecosystem using SNP markers
Source: PeerJ. 2026 Jan 13;14:e20580. doi: 10.7717/peerj.20580 (PMC12810364; doi:10.7717/peerj.20580)

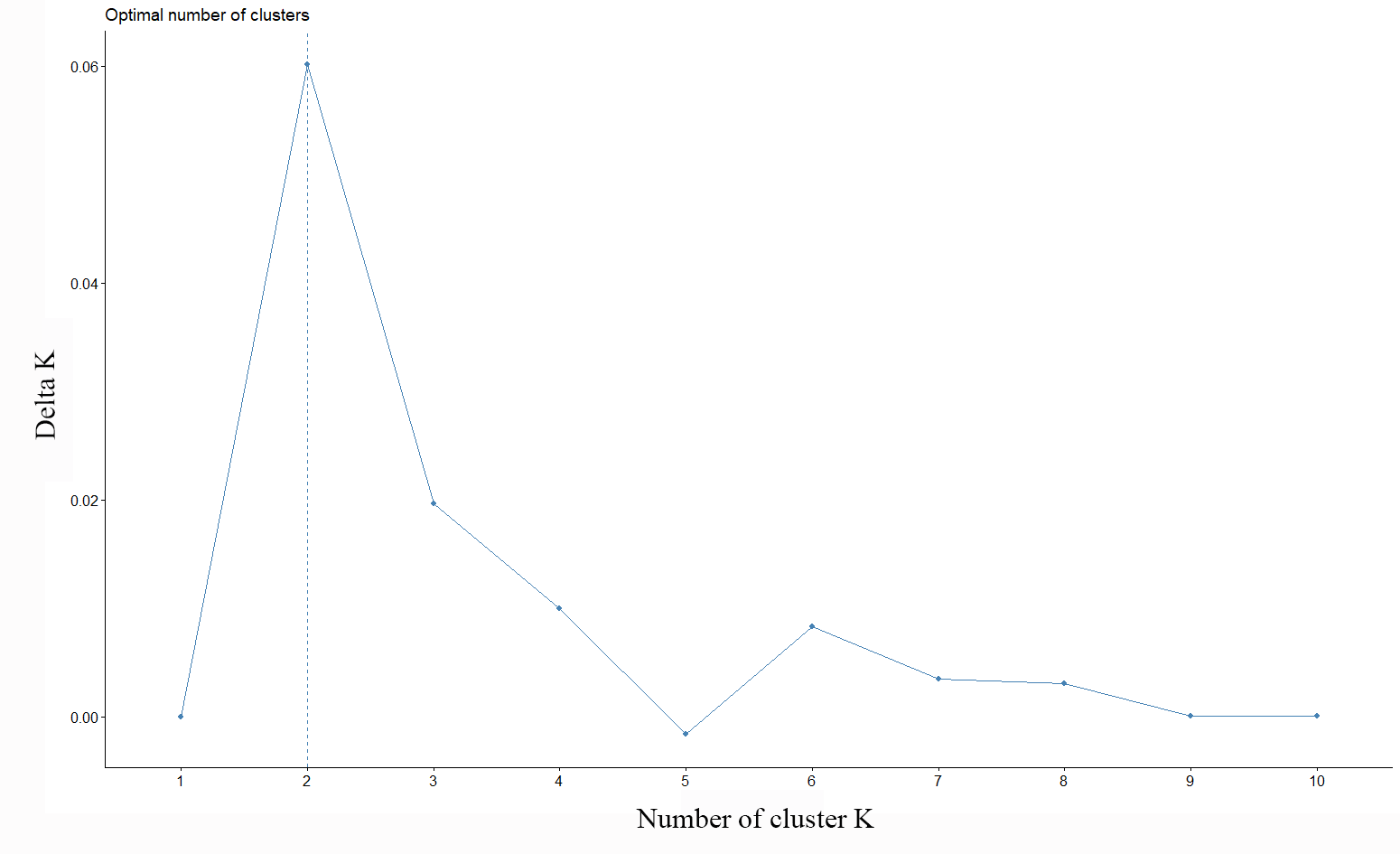

Supplement: Supplemental Information 4 [file peerj-14-20580-s004.png]
